# Mannitol oxidase and polyol dehydrogenases in the digestive gland of gastropods: Correlations with phylogeny and diet

**DOI:** 10.1371/journal.pone.0193078

**Published:** 2018-03-12

**Authors:** Alexandre Lobo-da-Cunha, Diogo Amaral-de-Carvalho, Elsa Oliveira, Ângela Alves, Vítor Costa, Gonçalo Calado

**Affiliations:** 1 Departamento de Microscopia, Instituto de Ciências Biomédicas Abel Salazar (ICBAS), Universidade do Porto, Porto, Portugal; 2 Centro Interdisciplinar de Investigação Marinha e Ambiental (CIIMAR), Matosinhos, Portugal; 3 Departamento de Biologia Molecular, Instituto de Ciências Biomédicas Abel Salazar (ICBAS), Universidade do Porto, Porto, Portugal; 4 Instituto de Investigação e Inovação em Saúde, Universidade do Porto, Porto, Portugal; 5 IBMC, Instituto de Biologia Molecular e Celular, Universidade do Porto, Porto, Portugal; 6 Departamento de Ciências da Vida, Universidade Lusófona de Humanidades e Tecnologias, Lisboa, Portugal; 7 MARE NOVA, Faculdade de Ciências e Tecnologia, Universidade Nova de Lisboa, Departamento de Ciências e Engenharia do Ambiente, Campus de Caparica, Caparica, Portugal; Waseda University, JAPAN

## Abstract

Mannitol oxidase and polyol dehydrogenases are enzymes that convert polyalcohols into sugars. Mannitol oxidase was previously investigated in terrestrial snails and slugs, being also present in a few aquatic gastropods. However, the overall distribution of this enzyme in the Gastropoda was not known. Polyol dehydrogenases are also poorly studied in gastropods and other mollusks. In this study, polyalcohol oxidase and dehydrogenase activities were assayed in the digestive gland of 26 species of gastropods, representing the clades Patellogastropoda, Neritimorpha, Vetigastropoda, Caenogastropoda and Heterobranchia. Marine, freshwater and terrestrial species, including herbivores and carnivores were analyzed. Ultrastructural observations were undertake in species possessing mannitol oxidase, in order to investigate the correlation between this enzyme and the presence of tubular structures known to be associated with it. Mannitol oxidase activity was detected in the digestive gland of herbivores from the clades Caenogastropoda and Heterobranchia, but not in any carnivores or in herbivores from the clades Patellogastropoda, Neritimorpha and Vetigastropoda. In most of the species used in this study, dehydrogenase activities were detected using both D-mannitol and D-sorbitol as substrates. Nevertheless, in some carnivores these activities were not detected with both polyalcohols. Ultrastructural observations revealed tubular structures in digestive gland cells of some species having mannitol oxidase activity, but they were not observed in others. Based on our results, we suggest that mannitol oxidase first occurred in a herbivorous or omnivorous ancestor of Apogastropoda, the clade formed by caenogastropods and heterobranchs, being subsequently lost in those species that shifted towards a carnivorous diet.

## Introduction

Mannitol oxidase and polyol dehydrogenases are enzymes that convert polyalcohols into sugars. Mannitol oxidase catalyzes the conversion of D-mannitol into D-mannose using molecular oxygen as hydrogen acceptor, releasing hydrogen peroxide [1]. This enzyme is known in terrestrial slugs and snails [2, 3], and in a few aquatic gastropods [4], but its overall distribution in the Gastropoda remained unknown. NAD^+^ is the hydrogen acceptor for polyol dehydrogenases, which are well known in animals, fungi, plants and prokaryotes. These enzymes belong to the medium-chain dehydrogenases/reductases (MDR) superfamily that include several alcohol dehydrogenases [5, 6]. Although sorbitol dehydrogenase was reported in the digestive gland of the freshwater snail *Viviparus viviparus* [7], polyol dehydrogenases are still poorly investigated in mollusks, the second largest phylum of multicellular animals. To contribute for filling these gaps in knowledge, we started by measuring oxidase and dehydrogenase activities in the digestive gland of several gastropods using mannitol and sorbitol as substrates.

Mannitol is a 6 carbon polyalcohol present in algae, fungi and plants, being one of the most abundant sugar alcohols in nature. In algae and plants this compound is a storage substance with great importance in osmoregulation that may also act as a scavenger of reactive oxygen species [8, 9, 10]. Thus, enzymes capable of converting mannitol and other polyalcohols present in algae and plants into sugars must be valuable for herbivorous gastropods [11]. Mannitol oxidase was first reported in the digestive gland and digestive tract of terrestrial snails and slugs [1, 2, 12] and in the freshwater snail *Biomphalaria glabrata* [11], all belonging to the clade Heterobranchia [13]. This enzyme was also detected by histochemical methods in the digestive gland of the marine heterobranch gastropods *Aplysia depilans* and *Siphonaria pectinata* [4, 14], but until now mannitol oxidase was not reported in gastropods outside the Heterobranchia. Mannitol oxidase was associated with a special kind of tubular structures, which form a cell fraction enriched in this enzyme in the digestive gland of land slugs and snails [3, 15]. Furthermore, biochemical studies showed that mannitol oxidase of terrestrial pulmonate gastropods can use other polyalcohols as substrates, presenting a higher activity with D-arabinitol and lower activity with D-sorbitol [2, 12]. On the other hand, sorbitol can be converted into fructose by sorbitol dehydrogenase, an enzyme that can also accept other polyalcohols as substrates, including D-mannitol [16], and an enzyme known as mannitol dehydrogenase was reported in bacteria, plants and fungi [9].

Among gastropods, as in other molluscs, the digestive gland is a large organ of the digestive system, being a suitable source of the enzymes under study. Typically, this gland is mainly formed by digestive and basophilic cells. Digestive cells are involved in endocytosis and intracellular digestion of food particles, whereas basophilic cells are considered responsible for the secretion of enzymes for extracellular digestion of ingested food. Detoxification, storage of minerals, accumulation of lipid and glycogen reserves are other functions of the digestive gland of gastropods [14, 17, 18, 19].

To obtain an overall view of the activity of mannitol oxidase and polyol dehydrogenases throughout the phylogenetic tree of gastropods, 26 species were investigated representing 22 families belonging to the clades Patellogastropoda, Neritimorpha, Vetigastropoda, Caenogastropoda and Heterobranchia. Besides taxonomic diversity, species living in marine, freshwater and terrestrial ecosystems, both herbivores and carnivores, were included to cover habitat and diet diversity. These herbivores include species feeding on microalgae, macroalgae or plants, whereas the carnivores include species eating protozoans or metazoans [20, 21]. Ultrastructural observations were undertake in species containing mannitol oxidase activity, in order to investigate the correlation between this enzyme and the presence of tubular structures in digestive gland cells.

## Material and methods

### Species and collection sites

Marine species were captured along the Portuguese coast (S1 Fig). Specimens of *Patella vulgata*, *Phorcus lineatus* (= *Monodonta lineata*), *Steromphala umbilicalis (= Gibbula umbilicalis)*, *Tritia reticulata (= Nassarius reticulatus)*, *Nucella lapillus*, *Ocenebra erinaceus*, *Aplysia depilans* and *Siphonaria pectinata* were collected on Porto city beaches (41.16° N, 8.68° W). Specimens of *Charonia lampas* were captured by local fishermen from Matosinhos (41.18° N, 8.70° W). Specimens of *Calliostoma zizyphinum* were collected on Aguda beach (41.05° N, 8.66° W). The common periwinkle, *Littorina littorea*, came from Ria de Aveiro a coastal lagoon connected to the Atlantic Ocean on the central coastline of Portugal (40.62° N, 8.74° W). The sea slugs *Aglaja tricolorata*, *Pleurobranchaea meckeli* and *Armina maculata* were collected on Tróia beach near Setubal (38.49° N, 8.90° W). Marine snails of the species *Hexaplex trunculus* were captured by local fishermen in the vicinity of the Culatra Island (36.98° N, 7.83° W) a barrier island of the Ria Formosa lagoon. The heterobranchs *Doriopsilla areolata* (Três Irmãos beach; 37.11° N, 8.56° W), *Bulla striata*, *Haminoea orbignyana* (Faro beach; 37.01° N, 7.99° W) and *Onchidella celtica* (Rocha beach; 37.10° N, 8.39° W) were collected on the South coast of Portugal. The freshwater snails *Neritina natalensis*, *Pomacea bridgesii* and *Marisa cornuarietis* were purchased in aquarium shops in Porto and *Planorbarius corneus* was provided by CEAR (Centro de Educação Ambiental das Ribeiras de Gaia, Vila Nova de Gaia). The terrestrial species *Cornu aspersum* (= *Helix aspersa*), *Lehmannia valentiana* and *Arion ater* were collected around Vila Nova de Gaia (41.12° N, 8.61° W). The taxonomic position, habitat and diet of these species, as well as the number of specimens utilized, are shown in Table 1.

**Table 1:** Taxonomy, habitat and diet of the 26 species included in this study. The number of animals of each species is indicated in brackets.

Gastropod clades				Family	Species	Habitat / Diet
**Patellogastropoda**				Patellidae	*Patella vulgata* (7)	Ma / Herbivore
**Neritimorpha**				Neritidae	*Neritina natalensis* (11)	Fw / Herbivore
**Vetigastropoda**				Trochidae	*Phorcus lineatus* (7)	Ma / Herbivore
					*Steromphala umbilicalis* (5)	Ma / Herbivore
				Calliostomatidae	*Calliostoma zizyphinum* (6)	Ma / Carnivore
**Caenogastropoda**	Hypsogastropoda			Nassariidae	*Tritia reticulata* (6)	Ma / Carnivore
				Ranellidae	*Charonia lampas* (6)	Ma / Carnivore
				Muricidae	*Nucella lapillus* (14)	Ma / Carnivore
					*Hexaplex trunculus* (6)	Ma / Carnivore
					*Ocenebra erinaceus* (6)	Ma / Carnivore
				Littorinidae	*Littorina littorea* (8)	Ma / Herbivore
				Ampullariidae	*Pomacea bridgesii* (7)	Fw / Herbivore
					*Marisa cornuarietis* (8)	Fw / Herbivore
**Heterobranchia**	Nudipleura	Pleurobranchomorpha		Pleurobranchidae	*Pleurobranchaea meckeli* (3)	Ma / Carnivore
		Nudibranchia	Doridacea	Dendrodorididae	*Doriopsilla areolata* (9)	Ma / Carnivore
			Cladobranchia	Arminidae	*Armina maculata* (5)	Ma / Carnivore
	Euopisthobranchia		Cephalaspidea	Aglajidae	*Aglaja tricolorata* (7)	Ma / Carnivore
				Haminoeidae	*Haminoea orbignyana* (7)	Ma / Herbivore
				Bullidae	*Bulla striata* (9)	Ma / Herbivore
			Anaspidea	Aplysiidea	*Aplysia depilans* (6)	Ma / Herbivore
	Panpulmonata			Siphonariidae	*Siphonaria pectinata* (7)	Ma / Herbivore
		Hygrophila		Planorbidae	*Planorbarius corneus* (14)	Fw / Herbivore
		Eupulmonata	Systellommatophora	Onchidiidae	*Onchidella celtica* (5)	Ma / Herbivore
			Stylommatophora	Helicidae	*Cornu aspersum* (8)	Te / Herbivore
				Arionidae	*Arion ater* (6)	Te / Omnivore
				Limacidae	*Lehmannia valentiana* (9)	Te / Herbivore

Ma—marine; Fw—freshwater; Te—terrestrial

No specific permissions for collection were required, because all marine and terrestrial species came from public areas not included in protected zones or national parks, and none of them are endangered or protected species. Freshwater species were breed in aquaria.

### Total homogenates for enzyme assays

Digestive gland homogenates were prepared in a Potter-Elvehjem homogenizer at 4°C, approximately with a tissue average concentration of 0.06 g.ml^-1^, using a medium for marine invertebrates containing 500 mM of sucrose, 150 mM KCl, 1 mM EDTA and 1 mM PMSF in 50 mM Tris-HCl buffer pH 7.4 (adapted from Moyers et al. [22] and Stewart et al. [23]). Homogenates were sonicated three times in cycles of 15 sec, with the tube plunged in an ice-water bath, using a Bandelin-Sonorex RK 100H bath sonicator. Samples were centrifuged at 1,000g for 5 min at 4°C, and the supernatants were used to assess enzyme activities shortly after being prepared.

Preliminary tests showed that a lower osmolarity medium containing 250 mM sucrose, 1 mM EDTA and 1 mM PMSF in 20 mM Tris-HCL buffer pH 7.4 (adapted from Moyers et al. [22]) did not improve the results obtained with non-marine species. Thus, since most species were marine, the high osmolarity medium was used for all species. Preliminary results also showed that 1% (v/v) Triton X-100 in the homogenates causes a considerable inhibition of mannitol oxidase activity in *A*. *depilans*. Although this effect was not detected in *C*. *aspersum*, sonication was used in all cases.

### Spectrophotometric enzyme assays

Mannitol oxidase activity was assayed spectrophotometrically by monitoring H_2_O_2_ production, following the method used by Cablé et al. [24] with adaptations based on Malik et al. [1]. The reaction medium contained: 1.06 mM phenol, 0.08 mM 4-aminoantipyrine, 5 U.ml^-1^ horseradish peroxidase, 0.06% (w/v) BSA and 25 mM of substrate (D-Mannitol or D-sorbitol), in 50 mM phosphate buffer pH 7.4. The reaction was started by the addition of 40 μl of tissue homogenate to 960 μl of reaction medium, and the increase of absorbance was measured at 500 nm. For control, non-specific increases of absorbance at 500 nm were monitored in medium without substrate, and subtracted when necessary. The production of H_2_O_2_ was calculated from the equation of a calibration line constructed using several standards of H_2_O_2_, as previously reported [25]. The catalase inhibitors sodium azide (10 mM) and 3-amino-1,2,4-triazole (50 mM), which were used by other investigators to prevent the interference of catalase [1, 26, 27], had a negative effect on the assessment of mannitol oxidase activity. Thus, these compounds were not used in this study.

Dehydrogenase activities were assessed spectrophotometrically by monitoring the formation of NADH at 340 nm (ε = 6.2 mM^-1^.cm^-1^). The assay media were prepared in 50 mM glycine/NaOH buffer pH 10.0, containing 5 mM NAD^+^ and 50 mM of the substrates D-mannitol or D-sorbitol (adapted from Fernandes et al. [28]). The reaction was started by the addition of 20 μl of tissue homogenate to 980 μl of assay medium.

Enzyme activities were measured at 25°C in a Jenway 6800 double-beam UV/vis. spectrophotometer connected to a water circulating system for temperature control in the cuvette. Except for *P*. *meckeli*, with only 3 specimens collected, for all the other species the average activities were obtained from individual measurements performed on 5 or more specimens. Results are expressed as nmol of product formed per min and per gram of digestive gland tissue (nmol.min^-1^.g^-1^).

### Detection of polyol dehydrogenases in native gel electrophoresis

Digestive gland homogenates of *P*. *vulgata*, *P*. *lineatus*, *L*. *littorea* and *S*. *pectinata* were centrifuged at 100,000 g for 1 h at 4°C. The supernatants (cytosolic fractions) were collected and 80 μg of protein (quantified according to Lowry et al. [29], using bovine serum albumin standards) were loaded in the wells of a 7.5% non-denaturant polyacrylamide gel. Electrophoresis was carried out for 4 h at 4°C. Subsequently, gels were sectioned to separate the lanes, which were incubated for about 3 h at room temperature in medium for detection of dehydrogenase activities with D-mannitol or D-sorbitol as substrates (adapted from Lewis and Gibson [30]). The incubation medium contained 0.01 mg.ml^-1^ phenazine methosulphate, 0.1 mg.ml^-1^ tetranitro blue tetrazolium chloride (TNBT), 3 mg.ml^-1^ NAD^+^ and 50 mM of polyol substrate in Tris-HCl 50 mM at pH 8.0 (TNBT precipitates at higher pH). Control lanes were incubated in medium without substrate. After washings in buffer, the sectioned gels were reassembled.

### Ultrastructural observations

Digestive gland samples were fixed for about 2 h at 4°C with 2.5% (v/v) glutaraldehyde and 4% (v/v) formaldehyde (obtained from hydrolysis of para-formaldehyde), diluted with cacodylate buffer pH 7.4, 0.4 M for marine species (final concentration 0.28 M) and 0.1M for non-marine species (final concentration 0.07 M). After washing with buffer, samples were postfixed with 2% OsO_4_ buffered with cacodylate, dehydrated in increasing concentrations of ethanol and embedded in Epon. Ultrathin sections were stained with uranyl acetate and lead citrate, before being observed in a JEOL 100CXII transmission electron microscope operated at 60 kV. For glycogen staining, ultrathin sections on copper grids were treated for 10 min with a 5% (w/v) solution of tannic acid, washed with water, and stained with uranyl acetate and lead citrate [31].

## Results

### Enzyme activities

Oxidase and dehydrogenase activities measured in digestive gland homogenates using D-mannitol or D-sorbitol as substrates are shown in Table 2. Mannitol oxidase activity was detected in the digestive gland of herbivores and omnivores belonging to the clades Caenogastropoda and Heterobranchia, but not in any carnivores or in herbivores belonging to the clades Patellogastropoda, Neritimorpha and Vetigastropoda. Mannitol oxidase activity was higher in the Eupulmonata (*O*. *celtica*, *C*. *aspersum*, *A*. *ater* and *L*. *valentiana*), with the highest average value in the land slug *A*. *ater*, which is considered an omnivore. The activity of this enzyme was also high in the marine slug *A*. *depilans*, but much lower in the freshwater snail *P*. *corneus* (Table 2). Oxidase activity with sorbitol was detectable in the digestive gland of Eupulmonata species, although being much lower than the activities with mannitol. Additionally, in the freshwater caenogastropods of the Ampullariidae family, represented by *M*. *cornuarietis* and *P*. *bridgesii*, a vestigial oxidase activity was barely detectable with sorbitol as substrate (Table 2).

**Table 2:** Oxidase and dehydrogenase activities in the digestive gland of gastropods. Values are mean (nmol.min^-1^.g^-1^) ± SD. Shaded lines correspond to carnivorous species, and dashes (**−**) denote undetected activities.

	Species	OXIDASE		DEHYDROGENASE	
		Mannitol	Sorbitol	Mannitol	Sorbitol
**Patellogastropoda**	*P*. *vulgata*	**−**	**−**	1,836.1 ± 368.6	1,644.3 ± 723.0
**Neritimorpha**	*N*. *natalensis*	**−**	**−**	870.2 ± 455.9	900.5 ± 356.4
**Vetigastropoda**	*P*. *lineatus*	**−**	**−**	1,214.4 ± 340.8	536.6 ± 211.2
	*S*. *umbilicalis*	**−**	**−**	1,322.9 ± 244.5	818.9 ± 320.0
	*C*. *zizyphinum*	**−**	**−**	**−**	**−**
**Caenogastropoda**	*T*. *reticulata*	**−**	**−**	**−**	**−**
	*C*. *lampas*	**−**	**−**	161.9 ± 60.4	418.4 ± 115.0
	*N*. *lapillus*	**−**	**−**	96.2 ± 97.1	381.4 ± 164.8
	*H*. *trunculus*	**−**	**−**	**−**	**−**
	*O*. *erinaceus*	**−**	**−**	109.1 ± 122.5	254.1 ± 332.5
	*L*. *littorea*	474.3 ± 148.3	**−**	510.2 ± 157.5	944.9 ± 293.8
	*P*. *bridgesii*	204.1 ± 59.0	17.6 ± 14.6	379.6 ± 65.3	741.0 ± 191.8
	*M*. *cornuarietis*	218.1 ± 138.8	6.6 ± 7.9	1,428.9 ± 232.1	4,095.5 ± 603.3
**Heterobranchia**	*P*. *meckeli*	**−**	**−**	**−**	60.7 ± 68.0
	*D*. *areolata*	**−**	**−**	202.0 ± 111.0	327.3 ± 141.9
	*A*. *maculata*	**−**	**−**	**−**	**−**
	*A*. *tricolorata*	**−**	**−**	133.6 ± 71.5	222.3 ± 89.4
	*H*. *orbignyana*	331.1 ± 234.4	**−**	224.0 ± 32.7	521.3 ± 139.5
	*B*. *striata*	126.6 ± 63.8	**−**	**−**	192.7 ± 82.6
	*A*. *depilans*	654.2 ± 107.9	**−**	131.5 ± 53.5	530.2 ± 150.1
	*S*. *pectinata*	239.4 ± 98.8	**−**	333.8 ± 116.3	460.2 ± 79.7
	*P*. *corneus*	68.4 ± 41.8	**−**	281.3 ± 140.7	847.9 ± 288.6
	*O*. *celtica*	1,468.5 ± 248.8	123.2 ± 48.3	623.8 ± 235.5	865.7 ± 157.4
	*C*. *aspersum*	722.6 ± 107.2	26.0 ± 44.5	546.2 ± 207.9	1,171.1 ± 335.8
	*A*. *ater*	1,722.4 ± 871.5	70.6 ± 30.1	651.6 ± 309.6	1,058.7 ± 501.1
	*L*. *valentiana*	1,422.4 ± 456.3	90.7 ± 58.5	437.4 ± 103.3	1,408.9 ± 319.2

Dehydrogenase activities were detected using both mannitol and sorbitol as substrates in most of the species included in this study. However, in the carnivorous *C*. *zizyphinum*, *T*. *reticulata*, *H*. *trunculus* and *A*. *maculata* dehydrogenase activities were not detected with both polyalcohols. Additionally, in the herbivore *B*. *striata* and in the carnivore *P*. *meckeli* dehydrogenase activity was not detectable with mannitol as substrate, and in these two species the activity with sorbitol had the lowest average values among the species in which sorbitol dehydrogenase activity was recorded. Moreover, in the carnivorous *N*. *lapillus* and *O*. *erinaceus* dehydrogenase activity with mannitol was not detected in some of the specimens, and the same occurred with sorbitol in *O*. *erinaceus* and *P*. *meckeli*. Because of that, the standard deviation in these cases is higher than the mean value (Table 2). Although the dehydrogenase activities with both polyalcohols were very variable among these species, there is a strong tendency for higher activities in herbivores than in carnivores. The highest sorbitol dehydrogenase activity was recorded in *M*. *cornuarietis*, being much lower in *P*. *brigdgesii* although these two species belong to the same family. Among herbivores lacking mannitol oxidase, dehydrogenase activities with mannitol were identical or higher than with sorbitol, while in herbivorous caenogastropods and heterobranchs the opposite tendency was observed. Carnivores in which polyol dehydrogenase activities were detected also showed a tendency to higher activities with sorbitol than with mannitol (Table 2).

**Fig 1:**
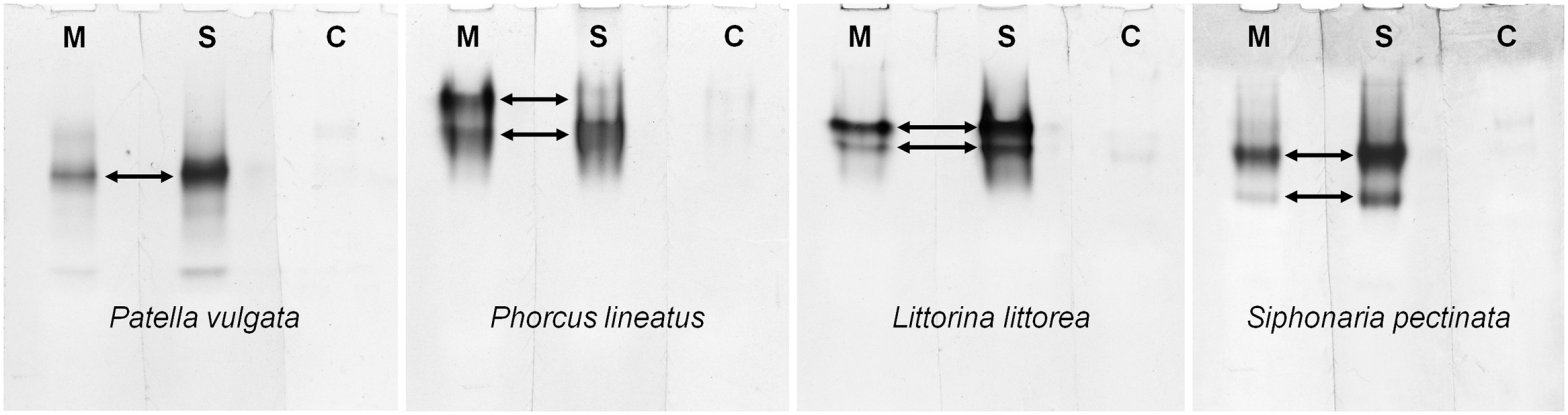
Analysis of dehydrogenase activities for the substrates D-mannitol (M) and D-sorbitol (S), in four species of marine gastropods. Proteins were separated by native gel electrophoresis and enzyme activity was assessed as described in methods. In controls without substrate (C) band intensities were negligible.

Enzyme activities were also analyzed *in situ*, after native gel electrophoresis, to find out if one or more proteins were responsible for the dehydrogenase activities detected with mannitol and sorbitol. For this preliminary analysis of the proteins involved in polyol dehydrogenation in gastropods, just one species from each of the four major gastropod clades (Patellogastropoda, Vetigastropoda, Caenogastropoda and Heterobranchia) was selected. This approach revealed one or two protein bands with polyol dehydrogenase activity in the digestive gland of the gastropod species that were analyzed. In the common limpet, *P*. *vulgata*, just one band revealed significant activity with both substrates. In *P*. *lineatus* only one band showed activity with sorbitol, whereas two bands showed mannitol dehydrogenase activity. The lower band with mannitol dehydrogenase activity seems to correspond to the enzyme with sorbitol dehydrogenase activity (since both bands have a similar relative mobility). In *L*. *littorea* and *S*. *pectinata* two bands revealed activity with both polyalcohols, but dehydrogenase activity was always higher with sorbitol (Fig 1).

### Ultrastructural observations

**Fig 2:**
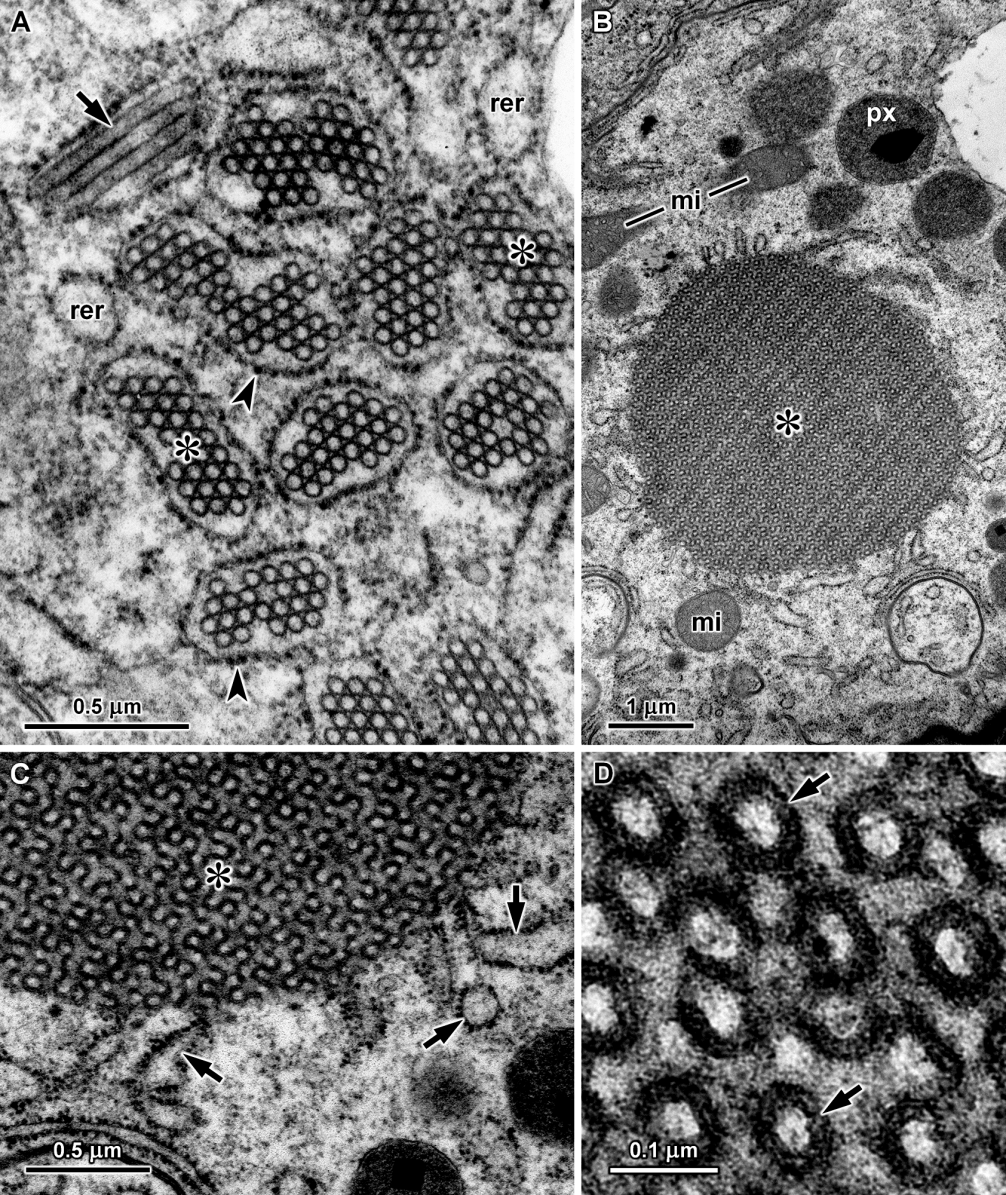
Tubular structures in the digestive gland of *A*. *depilans* (A) and *B*. *striata* (B-D). A. Tubular structures in transverse (asterisks) and longitudinal sections (arrow) within rough endoplasmic reticulum cisternae of a basophilic cell in *A*. *depilans*; arrowhead—ribosomes, rer—rough endoplasmic reticulum cisternae without tubules. B. Large aggregation of tubules in a basophilic cell of *B*. *striata*; mi—mitochondria, px—peroxisome. C. Rough endoplasmic reticulum cisternae (arrows) are associated with the margin of these aggregates of tubules. D. At high magnification it can be seen that the tubules of *B*. *striata* possess a double wall (arrows).

**Fig 3:**
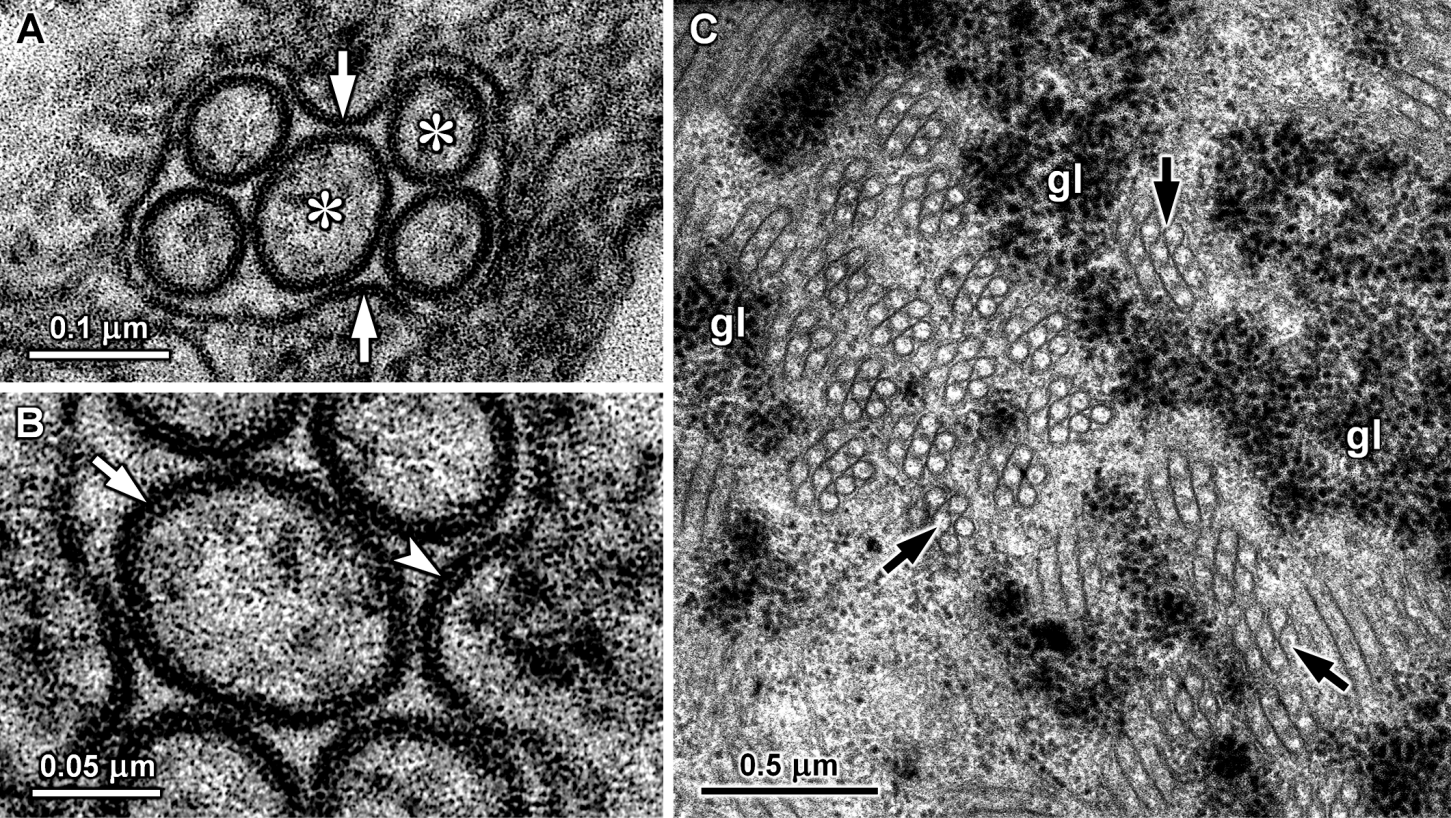
Tubules in digestive gland cells of *C*. *aspersum* (A-B) and *L*. *valentiana* (C). A. Transverse section of a smooth membrane cisternae containing tubular structures (asterisks) in *C*. *aspersum*. Infolds (arrows) can be seen in the membrane of the cistern. B. Detail of the previous section showing a thicker internal leaflet on the membrane infold (arrowhead), being similar to the outer leaflet of the tubule wall (arrow). C. In *L*. *valentiana*, tubule containing cisternae (arrows) were frequently associated with glycogen deposits (gl). Tannic acid was used to increase the electron density of glycogen particles.

Tubular structures were observed in digestive gland cells of *A*. *depilans*, *B*. *striata*, *C*. *aspersum*, *A*. *ater* and *L*. *valentiana* (Figs 2 and 3), but not in other species also containing mannitol oxidase, such as *H*. *orbignyana*, *S*. *pectinata* and *O*. *celtica*. In the basophilic cells of *A*. *depilans* many tubules with approximately 50 nm in diameter were seen inside several rough endoplasmic reticulum cisternae (Fig 2A), whereas in *B*. *striata* basophilic cells large aggregates of tubules were found in the cytoplasm with some rough endoplasmic reticulum cisternae at the edge (Fig 2B and 2C). At high magnification it could be seen that in *B*. *striata* the tubules had a double wall, approximately with an external diameter of 75 nm and an inner diameter of 45 nm. The lumen of these tubules was more electron-lucent than the substance around them (Fig 2D). Double-walled tubules were not observed in the other species. In *C*. *aspersum*, *A*. *ater* and *L*. *valentiana* the tubules were inside smooth membrane cisternae. Especially in *C*. *aspersum*, the membrane of some tubule containing cisternae presented infolds with a width similar to the diameter of the tubules inside the cisternae (Fig 3A). At high magnification it was possible to see that the cistern membrane was somewhat thicker in these infolds, with a thicker electron-dense internal leaflet resembling the wall of the tubules (Fig 3B). In *L*. *valentiana*, the cisternae with tubules were much more abundant at the basal region of digestive cells, being frequently surrounded by glycogen deposits (Fig 3C).

## Discussion

### Enzyme activities

**Fig 4:**
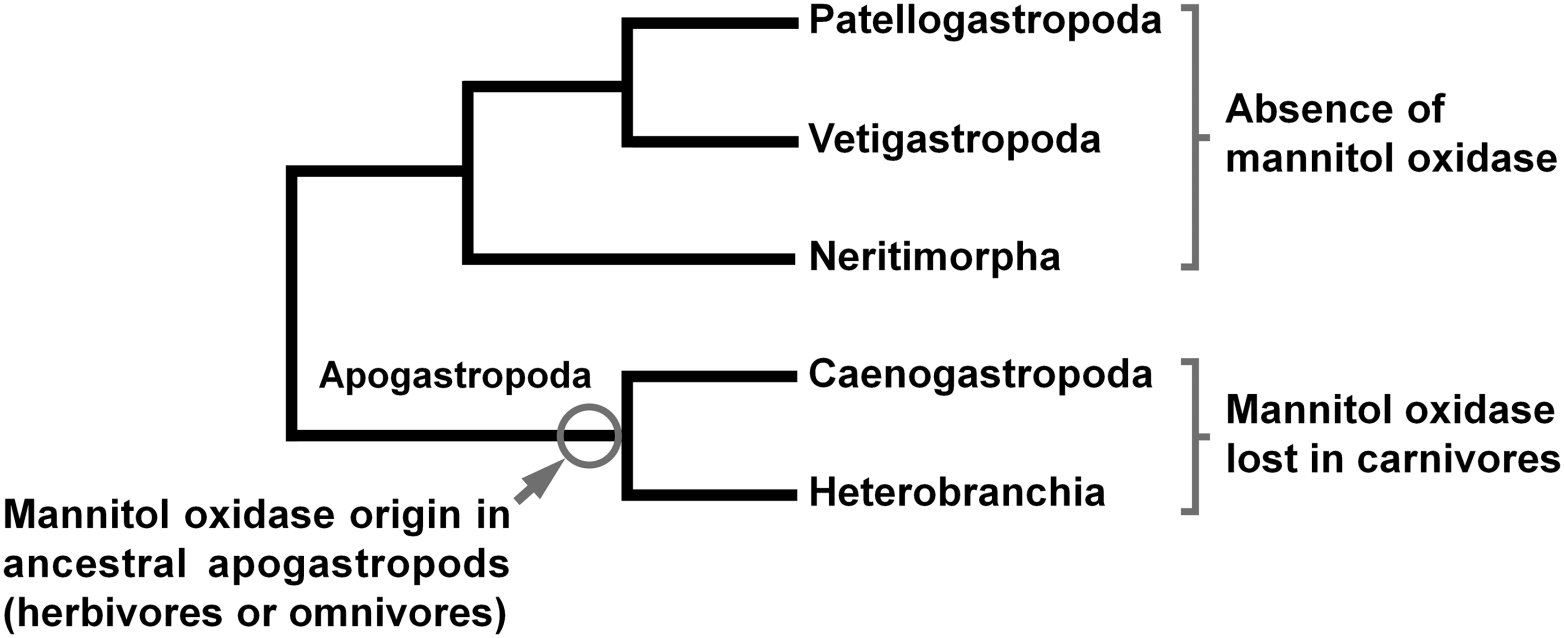
Hypothesis about mannitol oxidase evolution and distribution in the phylogenetic tree of gastropods. Phylogenetic tree according to one of the alternatives presented by Zapata et al. [32].

In gastropods, previous studies about polyalcohol oxidizing enzymes were restricted to few clades. Thus, the distribution of these enzymes in the gastropod phylogenetic tree was not previously described. The data obtained with the 26 species included in this study show that mannitol oxidase is present in herbivorous and omnivorous caenogastropods and heterobranchs, being absent in the clades Patellogastropoda, Vetigastropoda and Neritimorpha. Unfortunately, data about this enzyme in the deep-sea cocculinids and hot-vent gastropods are non-existent. Nevertheless, considering the current knowledge about gastropod evolution and phylogeny [13, 32], these results suggest that mannitol oxidase firstly occurred in a herbivore or omnivore ancestor of the Apogastropoda, the clade formed by caenogastropods and heterobranchs, being subsequently lost in those species that shifted to a carnivorous diet (Fig 4). The monophyly of Apogastropoda is well supported, although the exact phylogenetic relationships between the other major gastropod clades are not entirely solved [32]. However, since mannitol oxidase was found only in apogastropods, the position of the other clades in the phylogenetic tree of gastropods is not critical to this hypothesis. Although the clades Caenogastropoda and Heterobranchia include herbivores, carnivores and unselective feeders [33, 34], our data indicate that mannitol oxidase is absent only in carnivores. Therefore, our hypothesis about the origin of mannitol oxidase in gastropods suggests that the last common ancestor of all caenogastropods and heterobranchs was a herbivore or an unselective grazer containing mannitol oxidase. According to some authors, the ancestral gastropods were unselective grazers, with carnivory and herbivory evolving by gradual selection of vegetable or animal components of the food [35, 36]. In contrast to this view, herbivory (including grazing on microphytes) could have been the ancestral state, with carnivory evolving several times independently in different clades [34].

To date, it seems that mannitol oxidase is an enzyme exclusively present in mollusks. It was shown that the mannitol oxidase of terrestrial gastropods can also oxidize sorbitol, although with much less activity [2, 12]. Thus, the sorbitol oxidase activity detected in eupulmonates and Ampullariidae snails is, most probably, a residual activity of mannitol oxidase. Besides being well documented in gastropods, in the mussel *Mytilus galloprovincialis* mannitol oxidase activity was reported in gill epithelial cells, stomach epithelium and haemocytes, but not in the digestive gland [37]. However, in this mussel results were obtained using a histochemical method for cryosections and staining was equally observed in control sections incubated in medium without mannitol, being suppressed in heated sections [37]. Conversely, staining was not observed in control cryosections of other tissues incubated in medium without mannitol [4, 14, 37]. In others studies, staining in cryosections incubated in medium without substrate were considered false positive results [2, 4]. In our biochemical assays, reactions in medium without substrate were considered unspecific and subtracted from the values obtained with substrate. Nevertheless, the heat sensitive reaction reported in mussel tissues suggests an enzymatic reaction [37], but its exact nature still requires confirmation. Identification of the gene encoding mannitol oxidase would open an alternative way to detect this enzyme [38].

In herbivorous gastropods, enzymes capable of converting mannitol and other polyalcohols present in algae and plants into sugars must have significant nutritional value, increasing their fitness. Whereas mannitol oxidase seems to be restricted to herbivorous caenogastropods and heterobranchs, mannitol dehydrogenase activity was detected in all herbivorous species included in this study, except in *B*. *striata*. This means that herbivores belonging to the clades Patellogastropoda, Vetigastropoda and Neritimorpha are also capable of oxidizing mannitol. Moreover, with the exception of *B*. *striata*, species with mannitol oxidase apparently have two pathways to oxidize mannitol, because they also revealed mannitol dehydrogenase activity. In most species included in this study, dehydrogenase activities were detected with both mannitol and sorbitol. Considering that sorbitol dehydrogenase of mammals can use sorbitol, mannitol and other polyalcohols as substrates [16], polyol dehydrogenases of gastropods probably accept different substrates as well. Particularly in species in which mannitol oxidase activity is significantly higher to mannitol dehydrogenase activity (*B*. *striata*, *A*. *depilans*, *O*. *celtica*, *A*. *ater*, *L*. *valentiana*), it is likely that mannitol is mainly oxidized by mannitol oxidase while sorbitol is oxidized by a polyol dehydrogenase. Conversely, polyol dehydrogenases could be more relevant for the oxidation of mannitol in herbivorous gastropods in which mannitol oxidase activity is much lower than the dehydrogenase activity with this substrate (*M*. *cornuarietis*, *P*. *corneus*). Moreover, in herbivores without mannitol oxidase the oxidation of this polyalcohol will be totally dependent on polyol dehydrogenases and this could explain a tendency for higher dehydrogenase activities with mannitol in these species. In the carnivores, polyol dehydrogenase activities were not detected or were lower than in almost all herbivores included in this study, indicating that polyalcohol oxidation must be much less important in the metabolism of carnivorous gastropods.

Enzyme analysis *in situ*, after native gel electrophoresis, gave some preliminary information about the number of proteins that could be involved in polyol dehydrogenation in gastropods. In *P*. *lineatus*, two bands with mannitol dehydrogenase activity were detected, one of them corresponding to a protein that also exhibits activity for sorbitol. Thus, two polyol dehydrogenases seem to be present in this species, one with activity for both polyalcohols and another more specific for mannitol dehydrogenation. This could explain why in this herbivorous species lacking mannitol oxidase the dehydrogenase activity with mannitol was approximately two fold higher than with sorbitol. Conversely, in *P*. *vulgata*, in which dehydrogenase activity with mannitol was identical to the activity with sorbitol, these activities were detected only in one band. In *L*. *littorea* and *S*. *pectinata* two polyol dehydrogenase bands were also revealed after native gel electrophoresis, but all with higher activity for sorbitol. Thus, in these herbivorous species with mannitol oxidase it seems that polyol dehydrogenases are mostly involved in sorbitol metabolism. Nevertheless, further studies will be necessary to obtain a proper identification and characterization of these enzymes in gastropods.

### Tubular structures and mannitol oxidase

A particular kind of long tubular structures with a diameter about 50–60 nm were reported in the digestive gland, esophagus, crop and stomach epithelial cells of terrestrial pulmonate gastropods [39, 40]. Several studies using biochemical and histochemical methods showed that these tissues presented mannitol oxidase activity [2, 11], and cell fractionation methods revealed that these tubules constituted a fraction enriched in mannitol oxidase [3, 15]. In digestive gland cells of the land slug *Arion empiricorum* (= *A*. *ater*), Moya and Rallo [39] showed a continuity between the membrane of cisternae containing these tubules and the endoplasmic reticulum, and the same aspects were reported in *Arion lusitanicus* [40]. Additionally, in the digestive gland of the sea slug *A*. *depilans* bundles of these tubules were found inside rough endoplasmic reticulum cisternae [14]. Thus, taking into account both biochemical and ultrastructural data, mannitol oxidase carrying structures can be considered a specialized component of the endoplasmic reticulum, named mannosome [41].

In *B*. *striata* basophilic cells, rough endoplasmic reticulum cisternae were observed at the margins of the tubular arrays, but the tubules were never seen inside rough endoplasmic reticulum cisternae. The tubules of *B*. *striata* have a double wall and form compact aggregates in the cytoplasm, resembling the arrays of tubules with double wall and a diameter of 80–100 nm reported in esophageal epithelial cells of the land snail *Theba pisana* [42]. In the crop and stomach of this snail, isolated tubules with a double wall coexist with cisternae containing several tubules with a simple wall [42]. However, cisternae containing two or more tubules were never observed in *B*. *striata* basophilic cells. In *A*. *depilans*, the tubules have a simple wall and a diameter of 50 nm, while the double wall tubules of *B*. *striata* have an outer diameter about 75 nm. Nevertheless, excluding the outer layer of the double wall, the diameter of the inner tubule is close to 50 nm. This suggests that in *B*. *striata* each tubule was encircled by a membrane, creating a double wall, while in *A*. *depilans* and other gastropods several tubules with a simple wall are enclosed in a common cistern. Aggregates of tubules with a single wall, about 60 nm in diameter, associated with cisternae of rough endoplasmic reticulum were reported in digestive gland cells of the sacoglossan gastropod *Alderia modesta* that also feeds on algae [43]. These tubular aggregates resemble the ones observed in *B*. *striata*, although in *B*. *striata* the tubules had a double wall. However, in *A*. *modesta* a relationship between the tubules and mannitol oxidase activity was not investigated.

Based on ultrastructural images, Triebskorn and Köhler [40] suggested that the membrane of the endoplasmic reticulum forms ridges towards the interior of the cisternae, which later detach to originate intracisternal tubules. In *C*. *aspersum*, the depressions found in the membrane of the cisternae containing the tubules support that hypothesis. In these depressions, the inner leaflet of the membrane seems to be thicker than in the remaining part of the membrane, being similar to the outer leaflet of the wall of the tubules. The concentration of mannitol oxidase enzyme in some areas of the membrane of these cisternae could lead to the formation of the depressions that subsequently give rise to the intracisternal tubules. Although these tubules were always reported in species known to possess mannitol oxidase activity, they were not seen in the digestive gland of some marine gastropods containing a high mannitol oxidase activity, such as *O*. *celtica*. In these species the enzyme could be located in the membrane of the endoplasmic reticulum or diluted in the lumen of the cisternae. Ultrastructural localization of mannitol oxidase with antibodies could be a way to investigate this hypothesis.

### Concluding remarks

This study gives the first overall view about the distribution of mannitol oxidase and polyol dehydrogenase activities in Gastropoda. Based on the available results, it is conceivable that herbivorous and omnivorous caenogastropods and heterobranchs inherited the mannitol oxidase gene from a common ancestor with this type of diet. The absence of this enzyme in carnivorous caenogastropods and heterobranchs can be explained by the loss or inactivation of this gene in species that shifted towards a carnivorous diet. Additionally, it was revealed that herbivores without mannitol oxidase, belonging to the clades Patellogastropoda, Neritimorpha and Vetigastropoda, present a high mannitol dehydrogenase activity. The current results also show that in general the oxidation of polyalcohols is much more important for herbivorous than for carnivorous gastropods. Despite mannitol oxidase being known to be associated with tubular structures located inside the endoplasmic reticulum, such tubules were not observed in digestive gland cells of some species with a high mannitol oxidase activity.

## Supporting information

S1 Fig Map of collection sites along the Portuguese coast. (TIF)
