# Supplementary figures and images for: Mannitol oxidase and polyol dehydrogenases in the digestive gland of gastropods: Correlations with phylogeny and diet
Source: PLoS One. 2018 Mar 12;13(3):e0193078. doi: 10.1371/journal.pone.0193078 (PMC5846779; doi:10.1371/journal.pone.0193078)

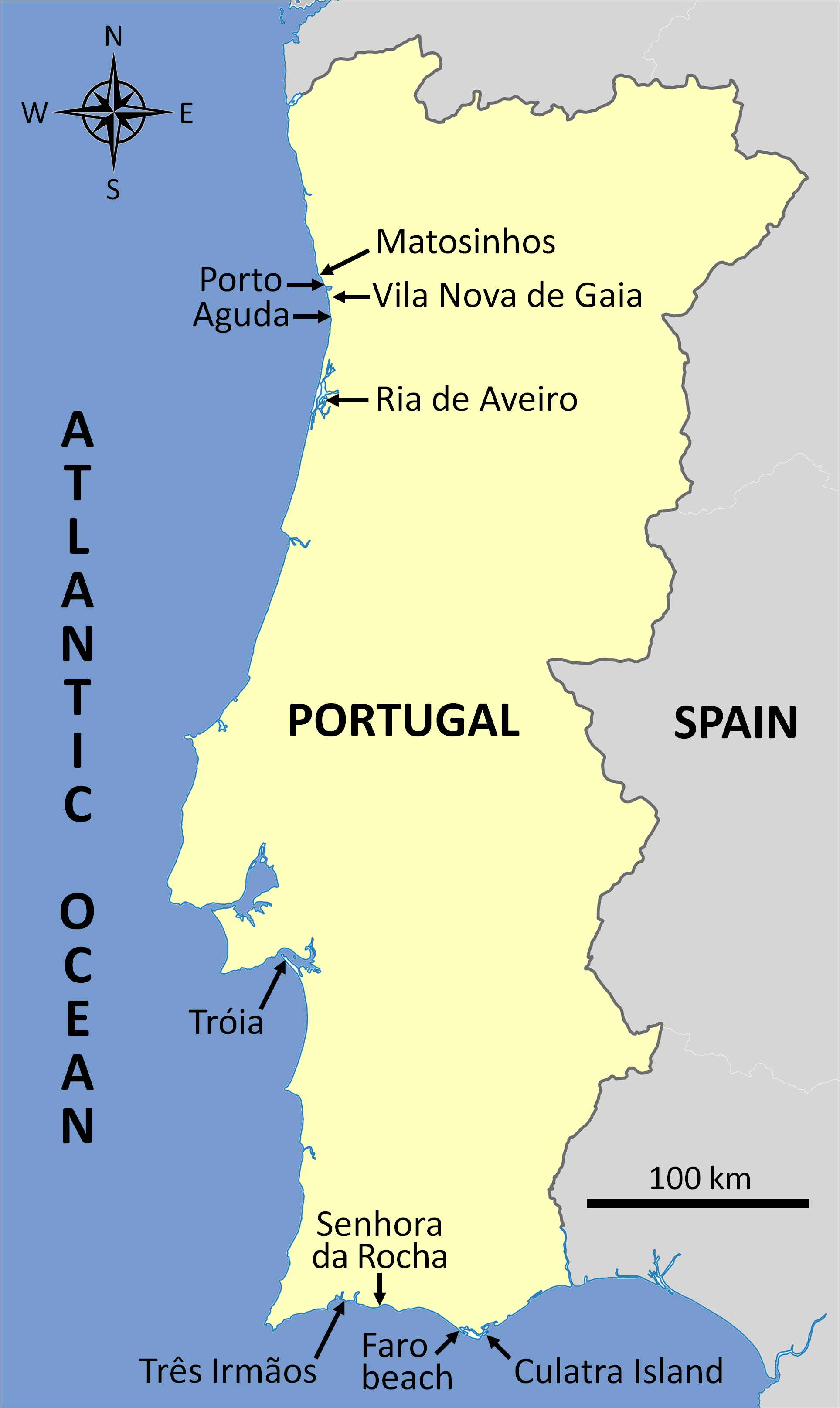

Supplement: S1 Fig — (TIF) [file pone.0193078.s001.tif]
